# Individual journeys to tuberculosis care in Nigeria’s private sector during the COVID-19 pandemic

**DOI:** 10.1136/bmjgh-2023-013124

**Published:** 2024-01-09

**Authors:** Charity Oga-Omenka, Lauren Rosapep, Elaine Baruwa, Lavanya Huria, Nathaly Aquilera Vasquez, Bolanle Olusola Faleye, Md.Abdullah Heel Kafi, Angelina Sassi, Chimdi Nwosu, Benjamin Johns, Abdu Adamu, Obioma Chijioke-Akaniro, Chukwuma Anyaike, Madhukar Pai

**Affiliations:** 1 School of Public Health Sciences, University of Waterloo, Waterloo, Ontario, Canada; 2 McGill International TB Center, McGill University Health Centre, Montreal, Vendôme, Canada; 3 ABT Associates Inc Bethesda, Bethesda, Maryland, USA; 4 Sustaining Health Outcomes through the Private Sector (SHOPS) Plus, Abuja, Nigeria; 5 Faculty of Medicine and Health Sciences, Epidemiology, Biostatistics, and Occupational Health, McGill University, Montreal, Quebec, Canada; 6 VizSight Analytics Inc, Vancouver, British Columbia, Canada; 7 National TB & Leprosy Control Program, Abuja, Nigeria

**Keywords:** COVID-19, tuberculosis, health services research, public health, treatment

## Abstract

**Background:**

Pre-COVID-19, individuals with tuberculosis (TB) in Nigeria were often underdiagnosed and untreated. TB services were mostly in the public sector with only 15% of new cases in 2019 reported from the private sector. Reports highlighted challenges in accessing care in the private sector, which accounted for 67% of all initial care-seeking. Our study examined patients’ health seeking pathways for TB in Nigeria’s private sector and explored any changes to care pathways during COVID-19.

**Methods:**

We conducted 180 cross-sectional surveys and 20 in-depth interviews with individuals having chest symptoms attending 18 high-volume private clinics and hospitals in Kano and Lagos States. Questions focused on sociodemographic characteristics, health-seeking behaviour, and pathways to care during the COVID-19 pandemic. All surveys and interviews were conducted in May 2021.

**Results:**

Most participants were male (111/180), with an average age of 37. Half (96/180) sought healthcare within a week of symptoms, while few (20/180) waited over 2 months. Individuals testing positive for TB had more health-seeking delays, and those testing negative for TB had more provider delays. On average, participants visited two providers in Kano and 1.69 in Lagos, with 61 of 180 in Kano and 48 of 180 in Lagos visiting other providers before the recruitment facility. Private providers were the initial encounters for most participants (60/180 in Kano, 83/180 in Lagos). Most respondents (164/180) experienced short-lived pandemic-related restrictions, affecting access to transportation, and closed facilities.

**Conclusions:**

This study showed a few challenges in accessing TB care, necessitating continued investment in healthcare infrastructure and resources, particularly in the private sector. Understanding the different care pathways and delays in care provides opportunities for targeted interventions to improve deployment of services closer to where patients first seek care.

**WHAT IS ALREADY KNOWN ON THIS TOPIC**

- Prior to the COVID-19 pandemic, TB in Nigeria was often underdiagnosed and untreated.
- The private sector accounted for 67% of initial care-seeking, but only 15% of new TB cases in 2019 were reported from this sector.
- Challenges in accessing TB care in the private sector have been reported in Nigeria.
- Previous studies have focused on health-seeking and provider delays in accessing TB care.
- Gaps in accessing TB care, including individuals who never access testing or are not diagnosed or treated, have been identified as major challenges in TB control in Nigeria.

**WHAT THIS STUDY ADDS**

- The study focused on individual TB health-seeking pathways in Nigeria’s private sector during the COVID-19 period.
- Most participants sought healthcare within a week of experiencing symptoms, but a significant proportion waited for over 2 months.
- Participants visited multiple healthcare providers before reaching the recruitment facility, with private providers being the initial encounters for most participants.
- Pandemic-related restrictions, including lockdowns, had a short-lived impact on TB care access.

**HOW THIS STUDY MIGHT AFFECT RESEARCH, PRACTICE OR POLICY**

- The study highlights challenges in accessing TB healthcare in Nigeria, even within a network of trained and supported private providers, emphasising the need for continued investment in healthcare infrastructure and resources, particularly in the private sector.
- Understanding the different care pathways and delays in care provides opportunities for targeted interventions to improve the deployment of services closer to where patients first seek care.
- The study underscores the critical role private healthcare providers could play to reduce delays in care-seeking and improve TB control in Nigeria and similar high-burden settings.

## Background

Before the COVID-19 pandemic, tuberculosis (TB) was the leading cause of infectious disease deaths globally.^1^ Of the 10.6 million estimated cases in 2021 worldwide, only 6.4 million (60%) were diagnosed and notified.^1^ Since the COVID-19 pandemic, global TB notifications and treatment have dropped, and deaths increased, erasing progress made in the last two decades.^1^ This decline suggests increases in community transmission and infections. Globally, several high-burden TB countries reported that the COVID-19 pandemic and control measures significantly impacted case notifications, treatment and other services for TB.^1–3^ The End TB Strategy calls for early diagnosis and treatment of TB,^4^ yet delays are persistent in many high-burden countries, particularly in the private sector.^5–8^ Extended delays are associated with increased risks of transmission, morbidity, and mortality.^9–12^

Nigeria, a high-burden TB country, had an estimated 467 000 incident TB cases in 2021, of which 208 000 were notified.^1^ TB case notifications in Nigeria, historically among the lowest in the world, have steadily improved from 17% in 2013, 24% in 2017 to 38% in 2020 and reaching 45% in 2021.^1 13^ Recent progress includes an increase in notifications from the private sector. However, these increases in private sector notifications remain relatively low, with only 15% of new TB cases reported from this sector in 2019. This discrepancy is noteworthy given that approximately 67% of initial TB care-seeking occurs within the private healthcare sector.^1 13 14^ The United States Agency for International Development (USAID)-funded project *Strengthening Health Outcomes through the Private Sector PLUS* (SHOPS Plus) aimed to strengthen the private sector’s capacity to detect and treat TB in alignment with international standards and the national TB control plan. By training and supporting over 2900 private providers, including clinical providers, laboratories, community pharmacists and proprietary patent medicine vendors in Lagos and Kano States, the project contributed to substantial increases in private sector TB notifications between 2017 and 2020.^15^

Nigeria recorded 88 388 confirmed cases of COVID-19 in 2020, with over 2 66 000 cases as of December 2022.^16^ During the initial lockdowns in March–April 2020, there were several reports indicating significant disruptions to TB services.^17–20^ Nigeria and other high-burden TB countries reported delays in care-seeking for respiratory symptoms due to fear of COVID-19 diagnosis.^19 21–24^ However, TB case-finding began to rebound in June 2020 and by the second wave in December 2020 to March 2021, TB notifications had fully recovered to pre-pandemic levels.^20^ This made Nigeria an outlier for pandemic recovery among other high-burden TB countries.^1^ However, it remains unclear how individuals navigate healthcare within Nigeria’s private healthcare sector, and if there are long-lasting impacts of COVID-19 on care-seeking patterns for chest symptoms.

Patient journeys or pathways analyses are useful approaches to understanding patients’ care-seeking patterns and how they access available services.^25 26^ These types of studies are designed to provide a retrospective account of patients’ healthcare journeys before reaching the facilities where they are finally diagnosed, with respect to numbers and types of facilities visited, delay by stage in pathway and factors associated with delays. Individual patient pathway analyses can provide insights on the number of attempts individuals make to find TB care and the missed opportunities for appropriate TB care. This information is necessary to surmount the problem of poor access to high-quality TB care and is key to finding the missing millions of TB cases that go undiagnosed globally every year.^1 25 27^ Pathway analyses have been used to highlight misalignment between demand and available resources at the macro level,^26^ or show the different care-seeking decisions at the micro level, as well as the types and numbers of providers that patients seek care with before getting diagnosed and treated.^28 29^ Several aspects of care pathways have been highlighted in literature, including time delays between symptom recognition, health seeking, diagnosis and treatment, as well as the number of healthcare encounters, type, and sector of providers encountered, and point of diagnosis.^26 29 30^

Using cross-sectional patient pathway surveys and qualitative case study interviews with individuals with diagnosed and presumptive TB recruited from facilities within the SHOPS Plus network, we aimed to assess how healthcare seeking and pathways may have changed in the Nigerian private sector during the COVID-19 pandemic.

## Methods

### Conceptual framework and definition of delays

**Figure 1:**
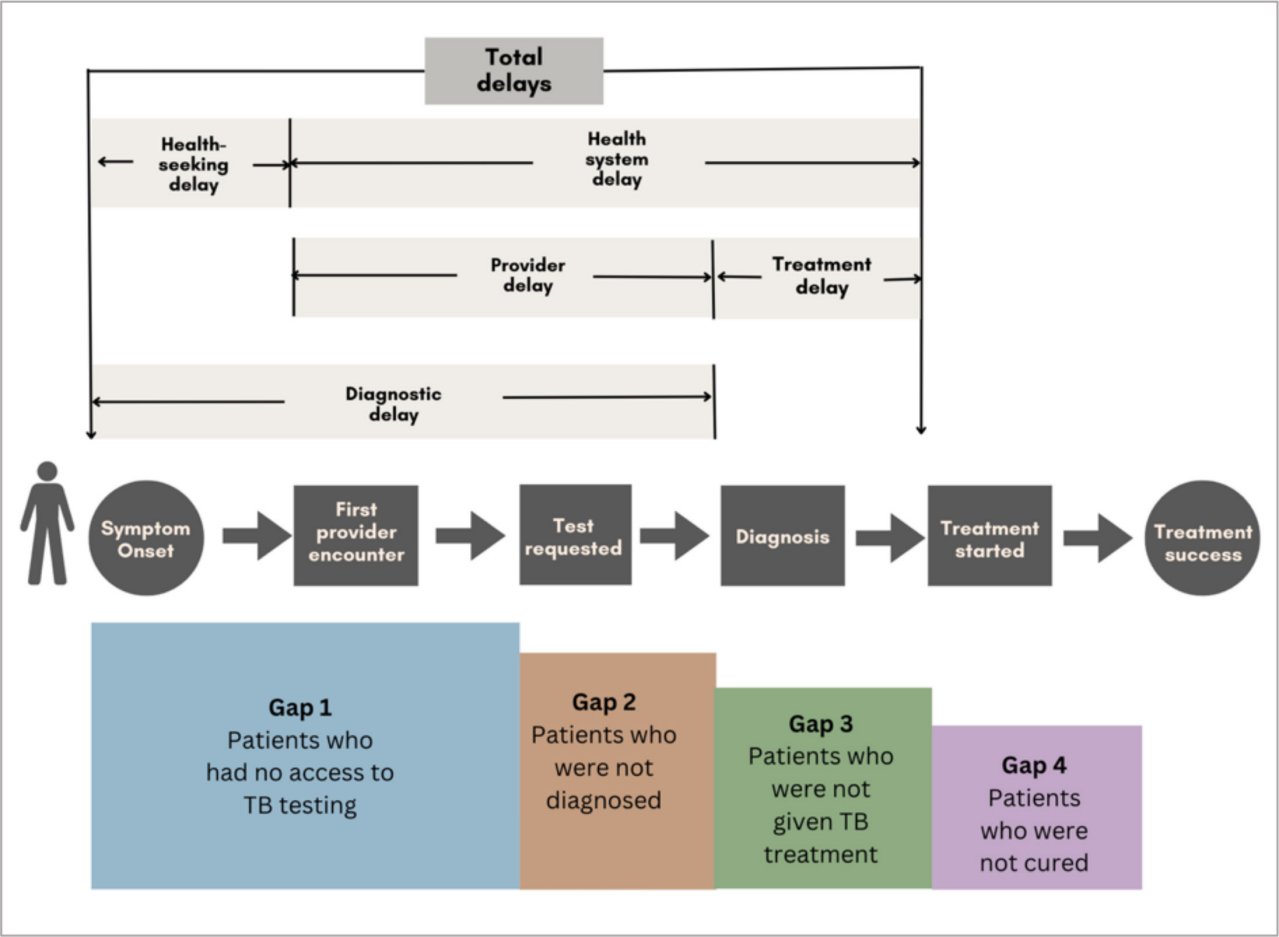
Conceptual framework on types of delays along the TB care cascade.^29 31–33^ TB, tuberculosis.

Our conceptual framework (figure 1) is derived from existing literature^29 31–33^ and highlights two main types of delays along pathways to TB care: health-seeking and health system delays. Health-seeking delays are defined as the time it takes from symptom onset to patients recognising their symptoms and seeking care from a healthcare provider. Health-seeking or patient delay, defined as the time from onset of treatment to the first healthcare visit,^31^ is influenced by lack of TB knowledge, stigma, cost of seeking care, among other factors.^34 35^ Health system delays occur once patients have sought care and can be further divided into provider delays and treatment delays.^35 36^ Provider or doctor delay is defined as the time between the first provider visit to when the patient is diagnosed.^31^ Provider delays occur when healthcare providers delay diagnosis due to low index of suspicion of TB, limited diagnostic tools, misdiagnosis or delays in making referrals, conducting tests or transmitting results.^31 35^ Treatment delay is defined as the time from diagnosis to the start of treatment.^31^ Treatment delays can occur when there is a lack of medication or equipment, insufficient staff or poor communication between providers and patients. Diagnostic delays refer to the time it takes from the onset of symptoms (individual-level) to the confirmation of TB diagnosis (health-system level). Lack of knowledge of TB (symptoms, diagnosis and treatment) is a common factor contributing to diagnostic delay.^37^

There is some consensus that to optimise patient outcomes, delays should not exceed 2–4 weeks for health seeking, 3–7 days for provider delay or testing using the current WHO-recommended TB diagnostics and 1–2 days for treatment initiation.^38–40^ The WHO considers as unethical any delay in treatment initiation in the presence of a positive diagnosis or a strong presumption of TB.^39^

Previous studies conducted in Lagos^37^ and rural Nigeria^6^ found that health-seeking delays were more common than provider delays, contributing more to overall total delay. The factors associated with these delays can be categorised as individual-level (knowledge, attitudes and behaviours) or structural-level (relating to healthcare facilities, providers, doctors, etc).

These delays can lead to three major gaps or missed opportunities in accessing TB care: Gap 1 includes those who never access TB testing, Gap 2 are those who access testing but are not diagnosed and Gap 3 includes those diagnosed but not treated. These gaps result in prolonged illness, disease transmission, increased risk of drug-resistant TB and death.^1 32 33^ Studies done in Nigeria before the pandemic identified Gap 1 as the biggest challenge in TB control, followed by Gap 2.^5 32 35 41^

### Study design, population and sampling

We conducted pathway analyses using mixed methods to survey 180 individuals and interview 20 individuals during the second COVID-19 wave in May 2021 (online supplemental appendix 1). This study was part of a larger research project in three high-burden countries. We previously published the timelines of the COVID-19 epi curve, TB notifications and the timing of our data collection in Nigeria.^20^ Our study took place among individuals seeking TB care in 18 private facilities in Kano and Lagos, the states with the highest TB burden and levels of private sector activity,^15^ and with estimated population of 14.7 million and 13 million, respectively, in 2021.^42^ We sampled active providers within the SHOPS Plus programme network: 1084 in Kano and 1317 in Lagos, out of which were 217 and 472 clinics or hospitals, respectively.

10.1136/bmjgh-2023-013124.supp1 Supplementary data

For the surveys, we used a non-probabilistic quota sampling to select the 18 highest-volume TB clinical facilities in the last quarter within the SHOPS Plus network (online supplemental appendix 2). This was done to ensure even geographic distribution of recruitment facilities within the states. We prioritised these facilities to minimise overall facility and network burden and increase recruitment efficiency. We included the three highest volume facilities (by total TB notifications) in each senatorial zone, ensuring only one facility per local government areas, resulting in a total of nine facilities per state.

We visited each consenting recruitment facility (RF) and developed a list of eligible patients from the facility’s National TB programme mandated presumptive and treatment registers, from which participants were randomly selected. Two types of patients were recruited—patients confirmed to be negative for TB (negative sputum test result in the register); and patients recently diagnosed (positive sputum test result for TB) receiving or scheduled for TB treatment within the same facility. All recruited patients were over 18 years old, HIV negative and had confirmed TB test results between January and May 2021 to minimise survey recall bias. We excluded previously confirmed COVID-19 cases, individuals unable to provide a sputum sample, patients initiating treatment at another facility, and those with missing sputum results or a history of anti-TB treatment in the past 6 months. Eligible individuals were contacted by facility staff to explain the study’s purpose and assess their initial willingness to participate. A date, time and place (the RF, patient’s home or place of work) were agreed on for the survey and interviews. Participants were also able to choose which of the three languages (English, Yoruba or Hausa) they wanted the questions in. We surveyed 10 participants in each RF.

For the qualitative interviews, we used a case study approach to conduct 1–2 additional in-depth interviews in each RF. Participants who consented asked to select location and preferred language for the interview.

### Data collection

Our study survey tool and interview guide focused on various aspects, including the onset of symptoms and care-seeking pathway, interactions with healthcare providers from initial encounters to TB diagnosis and treatment initiation, healthcare decision-making and utilisation during COVID-19, perspectives on the pandemic, and the impact of COVID-19 on patient care.

To ensure accuracy, the study tools were translated into local languages (Yoruba and Hausa), back translated to English to ensure fidelity to the original questions, pretested during a 3-day team workshop, and piloted in three facilities before final data collection. We also conducted 1 week data collection training per state for seven field officers each: three for the quantitative surveys, two for qualitative interviewing and two coordinators. Precautionary measures against COVID-19 were implemented prior to survey implementation, and verbal informed consent was obtained before interviews. Transport costs were reimbursed for participants who preferred interviews in the facility.

The survey instrument was programmed into SurveyCTO and uploaded to the server after completion.

We maximally varied qualitative interview participants by location, TB status and gender. Interviews, which lasted 24–55 min on average, were conducted in English, with respondents encouraged to request translation into or respond in Nigerian Pidgin, Yoruba or Hausa, if needed. All interviews were audio-recorded and transcribed, with translations from Yoruba and Hausa languages by fluent translators.

We used a framework approach involving both inductive and deductive thematic analysis.^31^ Codes were inductively derived and assigned to new themes or deductively derived from themes identified from an initial systematic review of barriers and facilitators to Drug Resistant (DR)-TB care.^32^ Interviews were coded by two graduate-level research assistants with additional training in qualitative research analysis (LH and NAV) and the first author (CO-O). All themes and codes were double-checked by CO-O. Other members of the research team checked the thematic analysis for overall alignment with study objectives. Transcripts were coded with aid of Quirkos software, V.1.6.1.

The research team included two seasoned scientists with expertise in social and TB research, along with a group of early to mid-career researchers boasting over 30 years of collective experience in TB programme implementation in Nigeria, many of whom were fluent in different languages used in data collection. The team also included three graduate research assistants, and two National TB programme officials. It is essential to note that none of the researchers played direct roles in managing patients with TB .

### Data analysis and pathway construction

The outcome variables were the number of encounters, and choice of first provider and delays. Our analysis involved descriptive statistics, construction of care pathways and logistic regressions. We weighted the data to address non-response and ensure the similarity between responders and non-responders in terms of background characteristics.^43 44^ We used logistic regression with covariates to calculate non-response weight, and predictive probabilities of survey response were calculated and derived. Unweighted mean of probabilities matched the 70% unweighted response rate in dataset.^3^ The non-response weight was determined by inverting the predictive probabilities,^44^ resulting in a sum equal to the sample size. We used descriptive statistics to analyse demographic characteristics of non-responders, considering both weighted and unweighted data. Univariate Pearson’s χ^2^ tests assessed associations between individual characteristics, TB status, private sector TB care delivery, impact of COVID-19, using weighted and unweighted cases.

We constructed individual patient-level pathways using stacked bar, which depict the chronological order in which individuals encountered various types of healthcare providers leading up to diagnosis and treatment. To create these sequencing charts, we accounted for points of diagnosis (including laboratories, community outreaches and traditional healers) even where the participants themselves did not acknowledge these as unique provider encounters.

Logistic regressions (weighted) were used to examine the relationship between patients’ characteristics and symptoms with the sector of first provider encountered after symptom onset, the number of provider encounters, as well as the use of multiple providers. We reported the OR with 95% CIs, considering a p value of <0.05 as statistically significant.

Statistical analysis was performed using the open-source programming language R (V.4.0.3) and R Studio (V.1.4.1106). Transcripts were coded inductively and deductively by two coauthors, CO-O and LH, using the Quirkos software (V.1.61), with an initial codebook developed from two transcripts. Codes and themes were double-checked by CO-O, and the thematic analysis reviewed by the research team for alignment with study objectives.

### Ethical considerations

Our study received ethical approval from the McGill University Health Centre (MUHC) Research Ethics Board (REB), with approval number Covid BMGF/2021-7197. We also obtained approval from the Health Research Ethics Committee (HREC) in both States, including from the Lagos State University Teaching Hospital (LASUTH) and Kano State Ministry of Health (MoH). We also obtained letters of introduction from Abt Associates to the MoH, National Association of Patent and Proprietary Medicines (NAPPMED), Association of Community Pharmacists in Nigeria (ACPN) and heads of the target facilities. All survey and interview participants gave written or verbal consent. We have included an author reflexivity statement on how we have ensured equity in our international collaborations as online supplement (online supplemental appendix 3).

### Patient and public involvement

Our research questions and data collection tools were developed considering previous studies in similar TB high-burden settings, and consultations with experts. Additionally, prior to recruitment, all survey and interview participants were given detailed information about the study and the included participants gave written or verbal consent. The study findings have been disseminated to the Nigeria National TB control programme and an online learning network of TB healthcare workers, researchers and other stakeholders from mostly high-burden countries.

## Results

### Characteristics of participants and non-response

Out of 337 patients contacted (online supplemental appendix 4), 180 participants completed the survey and 20 participated in the in-depth interviews. Higher non-response rates were observed among participants from Kano State compared with those from Lagos State (63% vs 38%) and those who tested negative for TB compared with those who tested positive for TB (54% vs 41%). Gender and age distributions were similar for both surveyed and non-surveyed participants, except for the 55+ age group, where non-surveyed rate was higher (19% vs 12%).

The participants (table 1) were predominantly male (62%), married (53%) especially in Kano State (58% vs 49% in Lagos), with an average age of 37 years. Participants in Kano had more dependent relatives on average (6) than in Lagos (3). Larger proportions were self-employed (43%), and had a secondary school education (34%). More participants lived within a 2 km radius from a public facility (48%), or a private facility (54%).

**Table 1:** Characteristics of surveyed participants by states (n=180)

	KANO	LAGOS
Gender		
Male	60 (66.7%)	51 (56.7%)
Female	30 (33.3%)	39 (43.3%)
Age		
Mean (SD)	35.9 (13.8)	38.5 (12.9)
TB status		
TB negative	45 (50.0%)	46 (51.1%)
TB positive	45 (50.0%)	44 (48.9%)
Marital status		
Married	52 (57.8%)	44 (48.9%)
Single	33 (36.7%)	36 (40.0%)
Widowed	2 (2.2%)	6 (6.7%)
Separated	0 (0%)	4 (4.4%)
Divorced	3 (3.3%)	0 (0%)
Number of dependent relatives		
Mean (SD)	5.77 (4.49)	2.77 (2.02)
Median (min, max)	5.00(0, 28.0)	3.00(0, 11.0)
Highest education		
Senior secondary	29 (32.2%)	32 (35.6%)
Primary six or lower	9 (10.0%)	20 (22.2%)
Bachelor’s or higher	7 (7.8%)	15 (16.7%)
OND/HND*	12 (13.3%)	5 (5.6%)
Junior secondary	3 (3.3%)	8 (8.9%)
Trade or vocational	2 (2.2%)	4 (4.4%)
Missing	28 (31.1%)	6 (6.7%)
Current employment		
Self-employed	45 (50.0%)	33 (36.7%)
Employee	19 (21.1%)	41 (45.6%)
Not working	26 (28.9%)	16 (17.8%)
Nearest public facility		
Less than 2 km	55 (61.1%)	31 (34.4%)
Between 2 and 10 km	32 (35.6%)	27 (30.0%)
Don’t know/not sure	1 (1.1%)	19 (21.1%)
Over 10 km	2 (2.2%)	13 (14.4%)

*OND/HND (representing 2–3 years postsecondary education.

HND, Higher National Diploma; OND, Ordinary National Diploma; TB, tuberculosis.

### Health-seeking and health system delays

Half of the 180 (n=96, 53%) participants sought care with a provider within a week of noticing their symptoms, with 18% seeking care within the first 2 days (table 2). Only very few patients (n=20, 11%) waited for 2 months and above before seeking care.

**Table 2:** Number of encounters, delays and the impact of COVID-19 on care-seeking

	Unweighted		Weighted		P value
	Kano	Lagos	Kano	Lagos	
Time from symptom onset to first provider encounter (health-seeking delay)					
1–7 days	49 (54.4%)	47 (52.2%)	52.3 (55.1%)	44.3 (52.9%)	0.102
1–4 weeks	21 (23.3%)	17 (18.9%)	22.0 (23.2)	15.8 (18.9)	
1–2 months	10 (11.1%)	16 (17.8%)	10.2 (10.8)	14.7 (17.57)	
2–3 months	5 (5.6%)	1 (1.1%)	5.2 (5.5)	0.9 (1.12)	
Over 3 months	5 (5.6%)	9 (10.0%)	5.2 (5.5)	8.0 (9.58)	
Sought care at more than one facility					
Yes	61 (67.8%)	48 (53.3%)	63.8 (67.3%)	44.0 (52.6%)	0.048
No	29 (32.2%)	42 (46.7%)	31.1 (32.7%)	39.7 (47.4%)	
Number of encounters†					
Mean (SD)	2 (0.936)	1.69 (0.76)	2.0 (0.9)	1.7 (0.75)	0.015
Median (min, max)	2(15)	2(14)	2(15)	2(14)	
Number of providers encountered†					
Only at recruitment facility	29 (25%)	42 (41%)	31.1 (32.7%)	39.7 (47.4%)	0.183
Two provider encounters	58 (50%)	46 (45%)	63.8 (67.3%)	44.0 (52.6%)	
Three provider encounters	21 (18%)	12 (12%)	21.7 (22.9%)	10.8 (12.9%)	
Four provider encounters	6 (5%)	2 (2%)	6.1 (6.4%)	1.8 (2.1%)	
Five provider encounters	2 (2%)	0 (0%)	2.0 (2.1%)	0	
First provider contacted after symptoms					
Private provider	60 (66.7%)	83 (92.3%)	63.3 (66.7%)	77.5 (92.5%)	0.001
Public provider	27 (30.0%)	5 (5.5%)	28.2 (29.4%)	4.5 (5.4%)	
Informal provider	22 (33.3%)	2 (42.2%)	3.3 (3.5%)	0.9 (1.1%)	
Testing with first provider contacted					
Provider did not discuss COVID-19	55 (61.1%)	40 (44.4%)	57.3 (60.4%)	36.8 (44.0%)	0.208
Provider did not discuss TB*	39 (43.3%)	32 (35.6%)	40.4 (42.6%)	29.4 (35.1%)	0.154
Provider said lab test was needed*	36 (40.0%)	22 (24.4%)	37.7 (39.7%)	20.1 (23.9%)	0.055
Provider recommended sputum test*	25 (27.8%)	14 (15.6%)	26.7 (28.1%)	12.8 (15.3%)	0.067
Time from TB diagnosis to treatment start (treatment delay)					
Same day or within 2 days	34 (37.8%)	32 (35.6%)	34.1 (60.4%)	28.8 (32.0%)	0.075
3–7 days	3 (3.3%)	10 (11.1%)	3.2 (3.5%)	9.1 10.1%)	
30 days	4 (4.4%)	2 (2.2%)	4.1 (4.6%)	1.9 (2.1%)	
2 months	2 (2.2%)	0 (0%)	1.9 (2.1%)	0 (0%)	
Over 2 months	2 (2.2%)	0 (0%)	2.2 (2.4%)	0 (0%)	
Negative for TB	45 (50.0%)	46 (51.1%)	49.4 (54.9%)	43.9 (48.8%)	
Impact of COVID-19 the health facility most frequented (multichoice)					
New infection control measures	46 (51.1%)	54 (60.0%)	48.7 (51.3%)	50.6 (60.4%)	0.222
Reduced hours	16 (17.8%)	3 (3.3%)	16.9 (17.8%)	2.69 (3.2%)	0.001
Screening for COVID-19	8 (8.9%)	4 (4.4%)	8.5 (8.9%)	3.63 (4.3%)	0.213
Closed	3 (3.3%)	2 (2.2%)	3.2 (3.4%)	1.81 (2.2%)	0.617
Since the pandemic in March 2020, has there been any time when you needed health care but did not receive it?					
No/cannot remember	84 (93.3%)	88 (97.8%)	88.4 (93.1%)	81.87 (97.8%)	0.130
Yes	6 (6.7%)	2 (2.2%)	6.5 (6.9%)	1.81 (2.2%)	
How COVID-19-related restrictions affected access to healthcare since March 2020 (multichoice)					
There has been no change for me.	57 (63.3%)	57 (63.3%)	59.8 (63.0%)	52.79 (63.1%)	0.996
Unable to leave the house to seek care because of movement/transport restrictions	20 (22.2%)	21 (23.3%)	21.1 (22.2%)	19.97 (23.9%)	0.792
Unable to reach a doctor because facilities were closed	15 (16.7%)	12 (13.3%)	15.7 (16.5%)	11.28 (13.5%)	0.574
Waiting times at facilities were longer than normal/expected	15 (16.7%)	12 (13.3%)	16.3 (17.1%)	11.35 (13.6%)	0.513
Unable to receive my medications because drug stores were closed	9 (10.0%)	6 (6.7%)	9.6 (10.1%)	5.72 (6.8%)	0.439
Sought care from a different facility than typical/preferred facility	8 (8.9%)	3 (3.3%)	8.5 (8.9%)	2.85 (3.4%)	0.130
How has COVID-19 and/or current/past COVID-19-related restrictions affected your willingness to seek healthcare?					
I am more willing to seek care	52 (57.8%)	66 (73.3%)	55.0 (57.9%)	61.02 (72.9%)	0.001
No effect	38 (42.2%)	18 (20%)	39.9 (42.0%)	17.13 (20.5%)	
Don’t know/not sure	2 (2.2%)	1 (1.1%)			
I am less willing to seek care	0 (0%)	6 (6.7%)	0	5.54 (6.6%)	

*These rates are calculated across as they form part of yes or no questions in the survey tool. Participants who answered ‘es’ to the question are shown in the table, as a fraction of total respondents to that question.

†The number of encounters used in the tables and regression were self-reported by the participants.

TB, tuberculosis.

**Figure 2:**
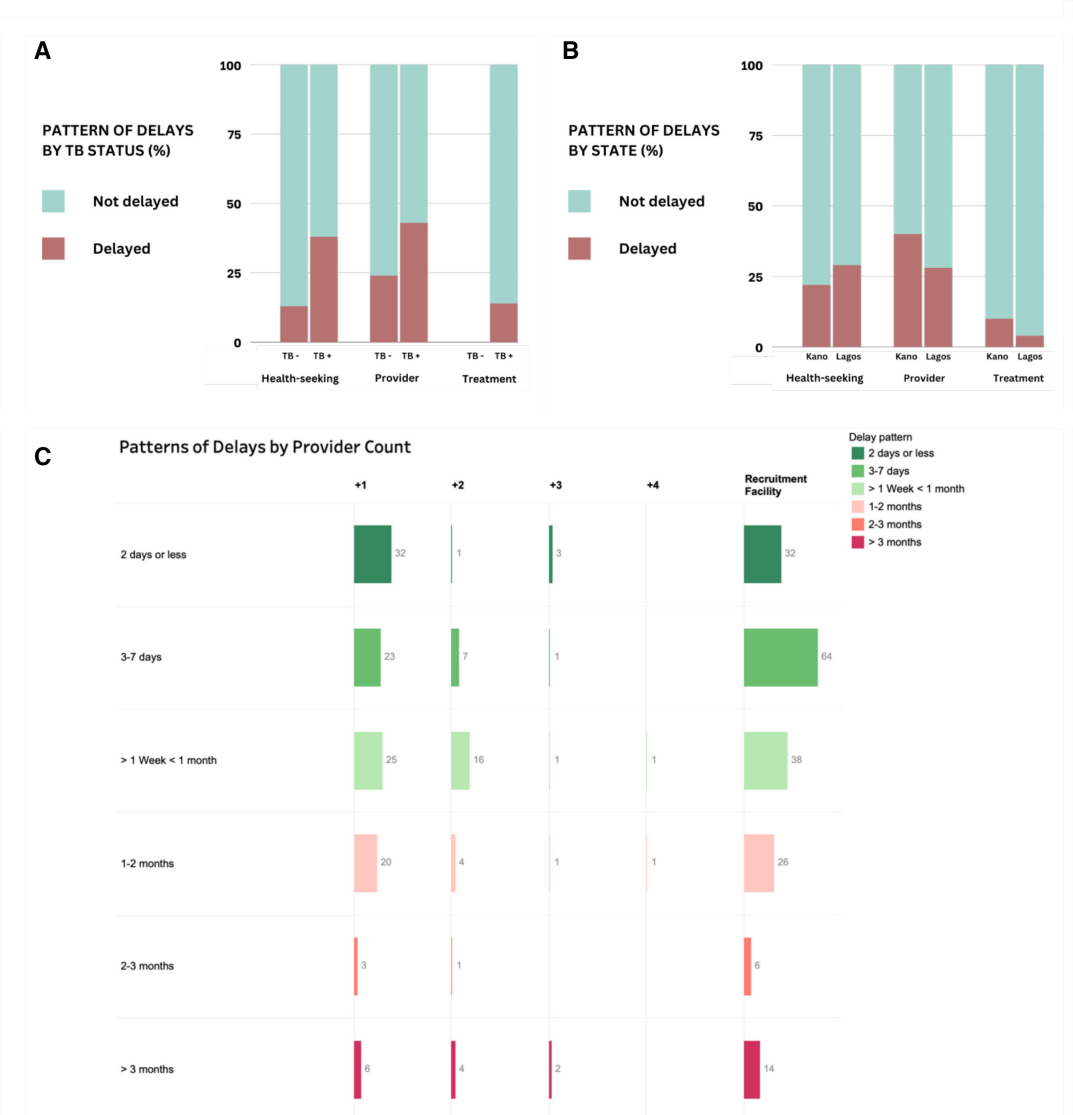
The proportion of participants experiencing different types of delays by (A) TB status, (B) State and (C) patterns of delays with each additional provider encounter for 180 participants. TB, tuberculosis

Health-seeking and provider delays were experienced more by participants diagnosed with TB (38% and 43% figure 2), with 14% of them experiencing treatment delays. Participants from Lagos (29%, figure 2) experienced more health-seeking delays. Provider and treatment delays were more in Kano (40% and 10%) compared with in Lagos (28% and 4%).

Out of 180 participants n=71 (39%) had their sole provider encounter at the study recruitment facilities (private clinics or hospitals), which included three individuals who had previously been diagnosed at community outreaches (table 2). Figure 2 presents the number of participants and the varying time delays at each provider encounter. Among 109 participants who visited other providers before the RF, 55 had their initial encounter within a week, 25 waited 1–4 weeks and 29 waited over a month. Out of the 33 who consulted two additional providers, 8 waited 1–7 days from the first encounter, 16 waited 1–4 weeks and 9 waited for over a month. Among eight who visited three providers, four waited 1–7 days following the second encounter, one waited 1–4 weeks and three waited for more than a month. Of the two who visited four providers, one waited 1–7 days, and the other waited 1–2 months.

At the RF, 96 of 180 participants waited 1–7 days, 38 waited 1–4 weeks and 46 waited more than 1 month. Of those who waited more than a month, the majority were individuals who had visited other providers. Delays between encounters increase with each additional provider encounter, particularly with the third and fourth encounters. Most patients with TB were initiated on treatment within 2 days of diagnosis (n=34, 75% in Kano; n=32, 73% in Lagos).

**Figure 3:**
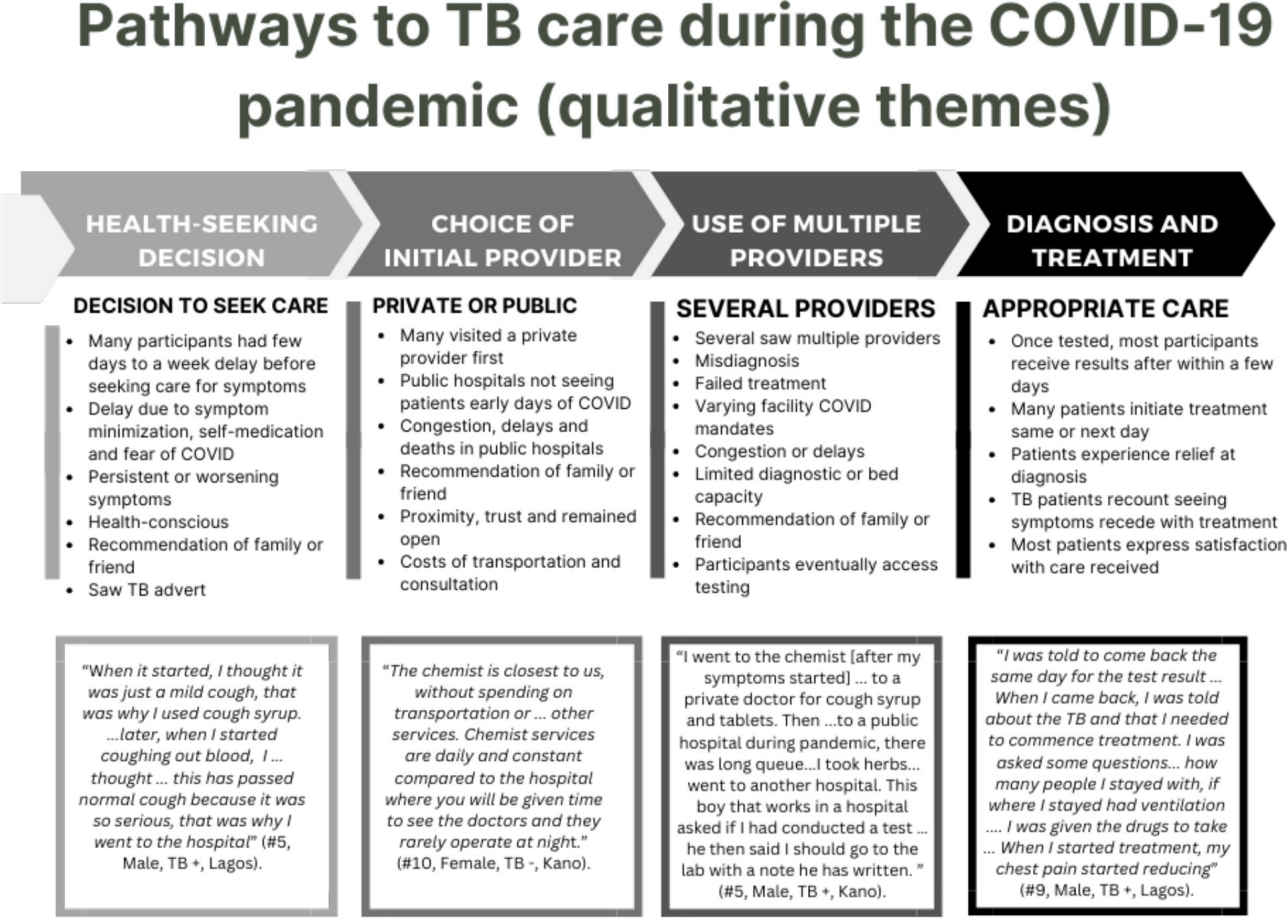
Qualitative themes on pathways to TB care. TB, tuberculosis.

From the qualitative interviews, many participants, regardless of TB status, reported health-seeking delays as they waited until their symptoms became prolonged. Symptom minimisation was a very common theme as most participants did not take coughing to be a serious health issue, at first (figure 3). Participants assumed that the cough would subside, and participants self-medicated. Some participants mentioned that they were also afraid of getting infected with or being diagnosed with COVID-19. Provider delays were mostly due to misdiagnosis and delayed referral to TB testing sites. All patients with TB interviewed in Kano and Lagos were initiated on treatment within 2 days of diagnosis.

### Individual patient pathways

On average, participants had two provider encounters in Kano, and 1.69 in Lagos, with a median of two in both States before visiting the RF (online supplemental appendix 5). Participants who visited the network of private hospitals and clinics had the fewest average encounters (mean of 1.10) compared with other providers (pharmacies or vendors=2.35, public clinic=3.00, traditional healers=3.00, private clinic=3.33). Our survey revealed that the majority (n=109, 61%) patients sought care from multiple providers. Twenty-five per cent of participants in Kano, and 41% in Lagos sought care only in the RF.

**Figure 4:**
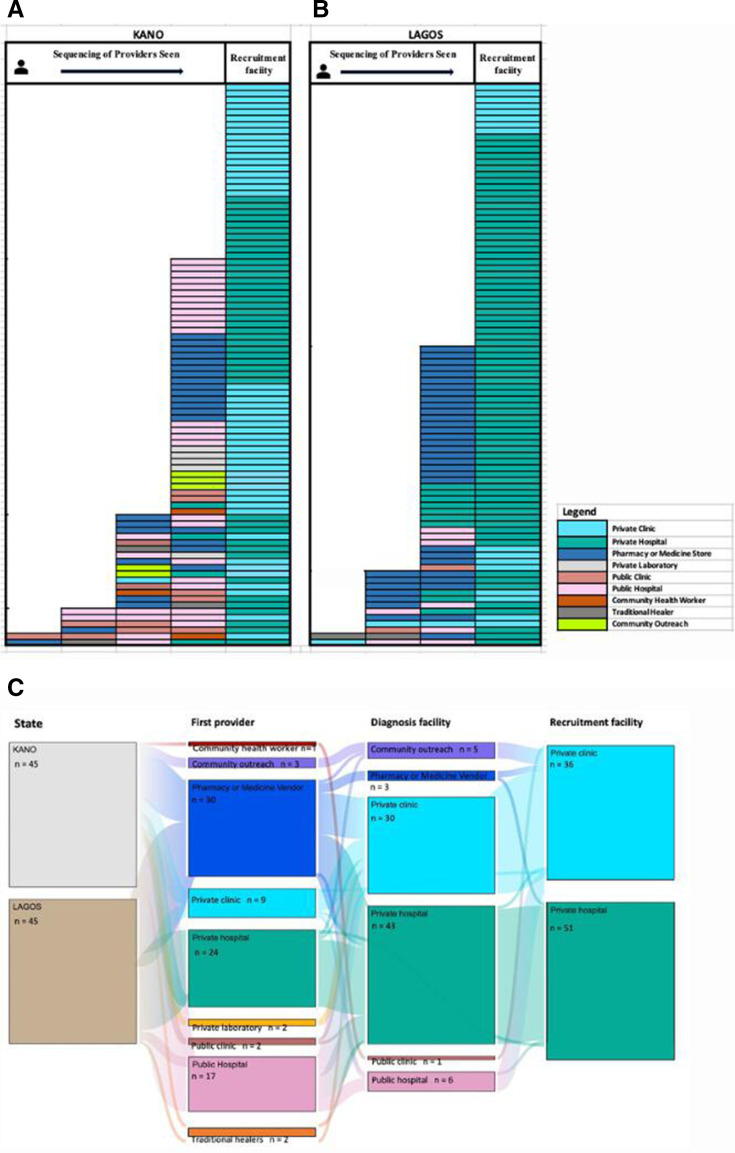
(A–C) Journey sequences for all survey participants in Kano (n=90) and Lagos (n=90). Sankey charts of participants who were positive for tuberculosis in Kano and Lagos.

Our pathway sequencing charts (figure 4a and b) identified 31 and 16 different patterns in Kano and Lagos, respectively, of progressive provider encounters. Participants in Kano, irrespective of their diagnosis, had more encounters and more variation in health-seeking than in Lagos. The largest share of participants in Kano used one additional provider (n=42, 47%), while for Lagos, the majority (n=42, 47%) used the RF exclusively.

Figure 4 shows pathway Sankey flow for all 90 survey participants diagnosed with TB and included points of diagnosis and type of RF. The majority of participants (n=73, 81%) were diagnosed in private clinics or hospitals, which were mostly the recruitment facilities. All participants (n=90) who were TB positive were initiated on treatment in the private hospitals and clinics that were our recruitment facilities.

The qualitative data (figure 3, online supplemental appendix 6) show many participants used a private provider first, because these providers were more accessible and convenient, and public hospitals were congested. In terms of choice of provider, this was either because of proximity, prior relationship with provider, cost considerations or due to referral or contact tracing from someone the participant knew. Several of those with more than one encounter said that the medicine store was their first port of call. Many participants visited multiple providers as their symptoms worsened, particularly those with TB. For participants diagnosed, once they were told they had TB, several said they were placed on treatment on the same or next day. Although the pathways to diagnosis were cumbersome for many, they expressed satisfaction with the care they received afterwards. The majority of themes regarding treatment were positive experiences after diagnosis.

### Impact of COVID-19

At the time of our survey, 74 (41%) participants said it was easier to access healthcare, while 46 (26%) said it was still difficult. Most respondents (164% or 91%) experienced lockdowns or other pandemic-related restrictions, which affected transportation to seek care for 41 (23%), closed facilities for 27 (15%) and longer waiting times for 27 (15%) of them. However, at the time of the survey, most respondents said they were more willing (118 or 66%) to seek healthcare, compared with before the pandemic.

From the in-depth interviews, early on in the pandemic, participants were concerned about the rising deaths due to COVID-19, which discouraged them from going to a clinic or hospital. Participants did not want to get infected, but more importantly, they did not want to be forced to take a diagnostic test for COVID-19, test positive, then have to quarantine, which was the practice in many public hospitals at the time. A few participants said transport challenges during the lockdown period hampered their access to care. Participants also mentioned provider attitudes during the early phase of the pandemic as being fearful or unhelpful.

### Determinants and factors influencing private sector use and number of encounters

We looked at predictors for two outcome variables: (1) choosing to use a private facility as their first healthcare contact; (2) using more than one provider from symptom onset. We also present the adjusted regression coefficient for the number of provider encounters.

**Figure 5:**
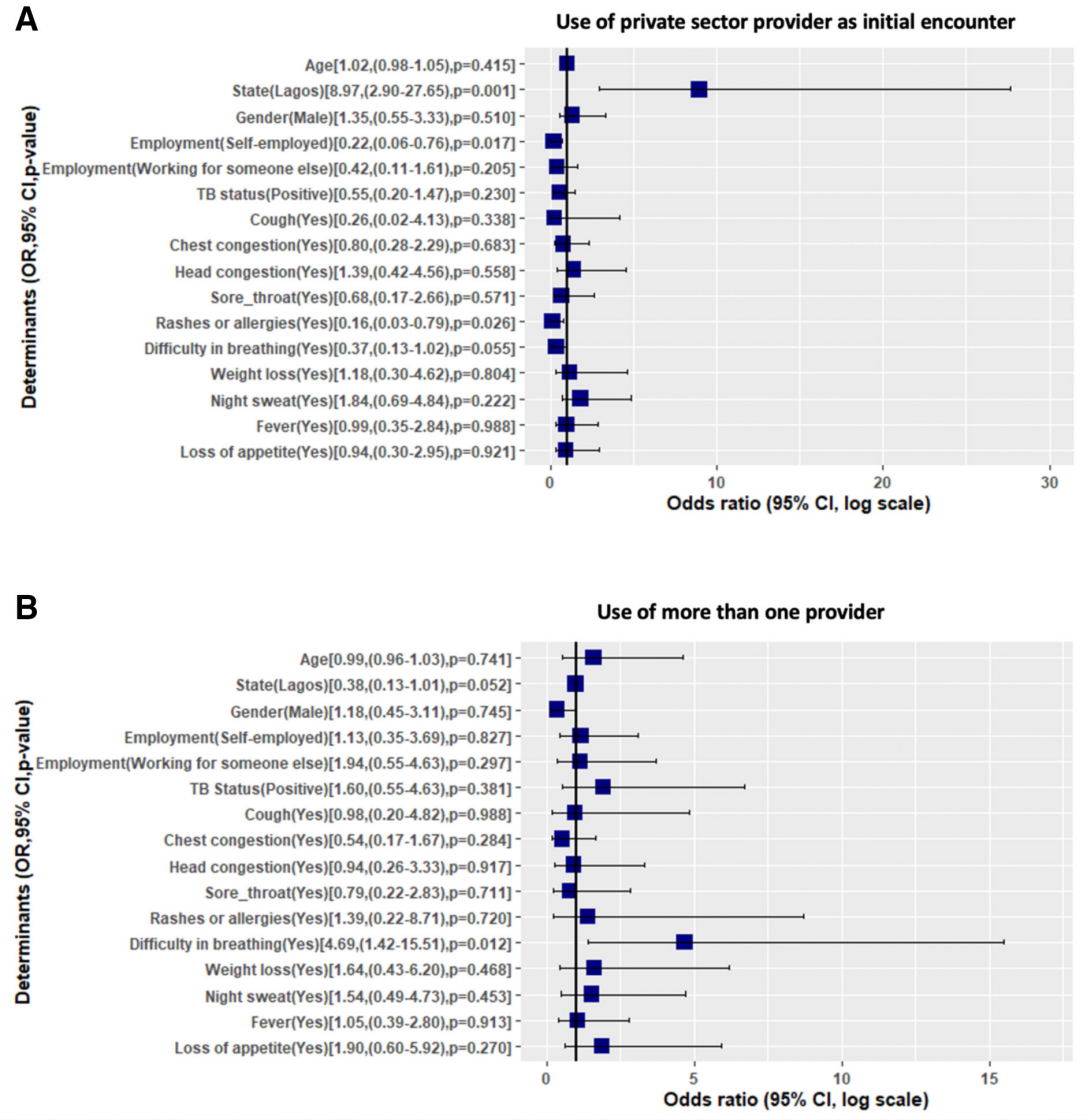
(A) Determinants of using the private sector as first provider contact. (B Determinants of using more than one provider after symptoms.

When we looked at the predictors for participants choosing to use a private facility as their first healthcare contact (figure 5A), we found that participants in Lagos State were more likely (OR=8.97, 95% CI: 2.90 to 27.65). Those who were self-employed (OR=0.22, 95% CI: 0.06 to 0.76), and those presenting with rashes and allergies (OR=0.16, 95% CI: 0.03 to 0.79), were less likely to use private providers first. No other variables, including age, gender, TB status or symptoms were significant. The only statistically significant determinant of using more than one provider (figure 5) was having difficulty in breathing as a symptom (OR=4.69, 95% CI: 1.42 to 15.51).

## Discussion

In our study of patient pathways within the private sector in two the Nigerian states, we found that individuals with chest symptoms experienced relatively short pathways. The study was conducted in a unique context, differing from the typical urban areas in Nigeria. The sampled providers belonged within the SHOPS Plus network, which provided extensive support to private sector facilities, ensuring efficient referral systems, availability of laboratory equipment and medications and community engagement for case identification. Even within this context, patients faced some health-seeking and provider delays.

### Health-seeking and health system delays

Our investigation showed several challenges associated with health-seeking and provider delays. However, once individuals with TB were identified and given a definitive diagnosis, their experience with the healthcare provider was much more positive and timelier than their experience before diagnosis, agreeing with several studies in Nigeria and other high-burden settings.^32 35 45^ Of the 90 participants who were TB positive, majority (n=66, 73%) and all of the qualitative interview participants were treated within 2 days of diagnosis, contributing to their feelings of relief or gratitude, because they finally felt, ‘seen’ by the healthcare system.

### Individual pathways and private sector use

Among the 180 participants, excluding the 71 who sought care first at the RF, the majority (74 out of 109) initially sought care in private facilities. Some studies in Nigeria and in similar high-burden TB countries have found that patients seek care first in drug stores or with traditional healers before clinics or hospitals,^6 37 46–48^ particularly if they were ‘only’ coughing.^47 49^

In our sample of networked providers, pharmacies and medicine vendors had higher rates of providing or referring patients for TB testing and treatment compared with clinics or hospitals, while the private hospitals and clinics provided appropriate care for every patient who visited them. This was due to the support this network of providers received, and the fact that most of the drug stores in our sample were located close to the private clinics and hospitals in the same network. Few studies have looked at the differences in referrals between different types of private providers; however, studies in Nigeria and in similar settings show that case notifications and referrals for TB care from the private sector have been historically very low and in need of intervention.^45–47 50–52^ The SHOPS Plus project, in collaboration with other stakeholders in Nigeria, have implemented several strategies to strengthen linkages between the private sector and the public TB delivery system.^53^

Several studies found that individuals with chest symptoms are very likely to minimise symptoms like cough or fever.^35 54–56^ Similar results have been reported in other high-burden TB settings where symptoms like weight loss were associated with faster care-seeking, in comparison to cough and fever which were perceived to be ‘normal’.^6 54 55 57^ In some settings, fever or headaches have been found to shorten delays.^58–60^

In charting patient pathways, our results show that all the patients who chose a private hospital (including RF) as their first or second provider did not use an additional provider. This was likely influenced by the fact that our recruitment facilities, which were supported sites, represented the first facility for 71 (40%) of all participants. We also found a similar pattern among patients who used a private hospital as second provider—of not going to another provider. These findings agree with studies from several high-burden TB countries, where hospitals, in public and private sector, missed diagnosing patients with TB.^52 61–63^ A pre-COVID-19 study in Nigeria found patients having up to five provider visits before reaching a provider within the National TB program (NTP) network.^64^

Qualitative themes related to misdiagnosis, lack of TB testing or referrals to other facilities were also common. Congestion and waiting times in health facilities also discouraged participants from seeking care, but that finding might have been a result of our purposive sampling in high-volume facilities.

While Nigeria has shown remarkable increases in number of annual case notifications,^1^ there are still approximately 208 000 missing cases estimated annually. Our findings are in concordance with several studies on TB care cascade showing that access to diagnostic services, or Gap 1, is the largest gap in the care cascade.^32 33 45 65^ Participants faced several barriers in accessing diagnostic services, especially during the start of the COVID-19 pandemic, and yet the responsibility for being diagnosed rests mostly on the patient, just like before the pandemic.^35^ Our findings agree with other studies showing participants who were TB positive face longer diagnostic delays,^32 33 35 66 67^ often due to lack of access to a diagnostic test.^32^

### Determinants of private sector use and numbers of providers

Our logistic regressions identified factors influencing private sector use and total number of providers encountered within the context of our study. Our participants were all recruited within a network of dedicated private providers in Kano and Lagos, making them distinct from the general population in terms of their inclination and capacity to use private healthcare. Participants in Lagos State were more likely to use the private sector first (93%) before going to the public sector, compared with 67% in Kano. This might be because self-employed individuals might be flexible with the time required to seek, and the lower cost of public healthcare compared with private healthcare. Two studies in India found young age, females, higher level of education and income group associated with private sector use.^68 69^ Several studies in Nigeria and other countries have suggested that patients are reluctant to use the public sector because of longer waiting times, sometimes poor quality of care, or poor provider attitudes, while on the contrary, private facilities have a reputation of better quality at a higher cost.^35 70 71^

Patients who had difficulty in breathing as a symptom were more likely to use more than one provider. Several studies have shown that patients with chest symptoms delay care-seeking for a variety of reasons and also contact several private providers before diagnosis.^72–75^ This is likely because of the symptom minimisation observed in several countries, where patients do not immediately seek hospital care for cough until symptoms deteriorate, preferring to self-medicate, or visit informal providers.^35 49 75–77^ The use of more than one provider has also been shown to be partly responsible for prolonged delays in TB diagnosis and treatment.^36 37 78 79^

### Impact of COVID-19

Participants in our study reported that the impact of COVID-19 on care-seeking was mostly felt during the lockdown periods, with a subsequent recovery to pre-pandemic levels. This finding aligns with the quick recovery of TB case notification in Nigeria compared with other countries.^1^ Disruptions to TB services have been widely documented in many high-burden settings.^24 80 81^ Lockdown and the movement restrictions posed barriers to accessing care, leading to delayed care-seeking due to difficulties in finding affordable transportation. Other concerns included the affordability of hospital care during the lockdown, fear of being diagnosed with COVID-19 or overburdened public hospitals. Some participants perceived their symptoms to be minor, further contributing to delayed care-seeking. Some participants narrated fearful healthcare workers’ attitudes towards them, particularly during the early days of the pandemic.

From the survey responses, most participants indicated that COVID-19 did not affect their care-seeking beyond the lockdown period. However, the qualitative data showed fear of COVID-19 was a predominant theme resulting in delayed care-seeking, although short-lived.

### Limitations

Our study has a few limitations. First, we were unable to calculate total provider delays across all provider encounters as our time measures were categorical and not exact dates. We also did not capture data on the total visits for each provider, as this would have provided a greater understanding of the total number of times participants sought care for their symptoms. Our analysis is limited by non-random purposive sampling for selecting facilities and patients and selection bias. We cannot say whether a study including patients diagnosed in the public sector or outside of the network of providers supported by the SHOPS Plus programme will show similar results. There was also no way to determine exactly if our sampled facilities were representative of the distribution of all facilities in Kano and Lagos. Finally, as patients were asked to recall events that had happened earlier, there might have been some recall bias in the responses. We attempted to mitigate this effect by only selecting recently tested patients, with encounters with the facilities dating back no further than January of 2021, or 5 months before our data collection. Additionally, the choice of higher volume facilities as recruitment facilities may have biased the results if high-volume facilities are better staffed to provide better care on average.

## Conclusion

Our patient pathway study in two major cities in Nigeria showed relative shorter individual pathways to care, given the unique context. It emphasises the importance of decentralising case identification and referral efforts, irrespective of the sector. Despite well-funded initiatives, delays persist, although potentially more manageable. Our study suggests that COVID-19 does not pose a lasting hurdle for the National TB control efforts among patients who are willing and able to use private healthcare. More patient education on the importance of getting tested with prolonged coughing, as well as improving referral systems and provider training within the private and public healthcare sectors are needed in our study settings. The WHO calls for multisectoral and intersectoral engagement in the fight against TB, and our findings support evidence from other high-burden settings that private sector provider engagement is critical.

## Data Availability

Data are available upon reasonable request. Data is available upon reasonable request. Data sharing agreements might be required prior to making the data available. Data include anonymised interview transcripts and survey responses.
