# Supplementary material for: Individual journeys to tuberculosis care in Nigeria’s private sector during the COVID-19 pandemic
Source: BMJ Glob Health. 2024 Jan 9;9(1):e013124. doi: 10.1136/bmjgh-2023-013124 (PMC10806899; doi:10.1136/bmjgh-2023-013124)
Supplement: Supplementary data [file bmjgh-2023-013124supp001.pdf]

BMJ Publishing Group Limited (BMJ) disclaims all liability and responsibility arising from any reliance placed on this supplemental material which has been supplied by the author(s)

Appendix

Appendix 1: Concurrent mixed methods and participant recruitment flow chart

Facility selection	**Sampling of recruitment facilities (n-18)**<br>1. Divided Lagos and Kano into 3 each by senatorial zones (n=6)<br>2. Selected 3 highest volume facilities based on notifications in the last 3 months from each senatorial zone, ensuring different local government locations (n =18)	
Participant recruitment	**Participant recruitment (337)**<br>1. TB client registers were reviewed starting from the most recent listings (within 6 months)<br>2. Clients over 18 years old and HIV negative with confirmed TB negative (presumptive) and TB positive (confirmed) sputum test results were included<br>3. Out of 337 patients contacted, 212 (63%) consented for the quantitative surveys, and 46 (14%) consented for qualitative interviews, and 77 (23%) were unreachable. Response rate = 77%.<br>4. Surveys were scheduled and conducted until quota (180 surveys) was reached.	
Data collection<br><br>*10th- 28th May 2021*	**Quantitative survey (n=180)**<br>1. In each state - 90 surveys (45 presumptive and 45 confirmed TB)<br>2. Questions on delays and pathways to TB testing and treatment and impact of COVID	**Qualitative interviews (n=20)**<br>1. In each state - 10 in-depth interviews<br>2. Questions explored facilitators and barriers to TB care initiation and impact of COVID
Data analysis	Descriptive statistics and logistic regression	Thematic coding
Results	**Integration of mixed methods results**<br>1. Statistically significant results were compared with qualitative themes<br>2. Mixed methods results were compared for complementarity and discordance	

Appendix 2: Quota sample distribution by site

State	Senatorial Zone	Facility Name	LGA	# of network facilities	Facility Type	Facility registers Jan – May 2021		Surveyed Jun 2021	
						TB negative	TB positive	TB negative	TB positive
Kano	Kano Central	Zakirai Nursing Home & Maternity	Dala	61	Hospital	187	56	5	5
	Kano Central	Kura Surgery and Maternity Clinic	Kura	14	Hospital	168	52	5	5
	Kano Central	International Hospital	Fagge	122	Hospital	447	50	5	5
	Kano Central	Al-Nouri Hospital	Tarauni	115	Hospital	515	31	5	5
	Kano North	Yakubu Dannono Memorial Hospital	Tofa	11	Hospital	318	39	5	5
	Kano North	Dawanau Clinic & Maternity	Dawakin Tofa	7	Hospital	152	3	5	5*
	Kano South	Al-Hilal Nursing & Maternity Home	Wudil	48	Nursing Home	244	69	5	5
	Kano South	AHIP FHC Clinic	Garko	29	Hospital	566	81	5	5
	Kano South	H. Nasiha Clinic and Maternity Centre	Gaya	45	Hospital	449	103	5	5
Lagos	Lagos Central	St. Lukes Hospital	Lagos Mainland	37	Hospital	75	14	5	5
	Lagos Central	Fenhitola Royal Consult	Lagos Island	63	Hospital	156	14	5	5
	Lagos Central	St. Kizito Clinic Lekki	Eti-Osa	30	Hospital	591	84	5	5
	Lagos East	Eloka Nursing Home	Ikorodu	72	Nursing Home	100	24	5	5
	Lagos East	Al Sadiq Hospital	Kosofe	46	Hospital	71	18	5	5
	Lagos East	R-Jolad Hospital	Shomolu	27	Hospital	179	12	5	5
	Lagos West	St. Theresa Hospital	Ajeromi-Ifelodun	113	Hospital	517	89	5	5
	Lagos West	Medservice Hospital	Alimosho	335	Hospital	58	23	5	5
	Lagos West	St Catherine Med.	Oshodi-Isolo	70	Hospital	115	19	5	5

** Sampled patients included 2 patients who were diagnosed within recruitment period earlier to complete quota*

Appendix 3 - Author Reflexivity Statement

1. **How does this study address local research and policy priorities?**

Nigeria, a high burden TB country, had an estimated 467,000 incident cases in 2021[1]. Case notification for TB in Nigeria has come mainly from the public sector. Only 15% of new TB cases in 2019 were reported from the private sector, even though it accounts for approximately 67% of initial TB care-seeking. Increasing TB notifications from the private sector is a priority for the Nigerian Government.

2. **How were local researchers involved in the study design?**

The research study was co-designed by the co-PIs (MP, JD, and EB), overseen by CO, and in collaboration with the SHOPS Plus project, including local researchers BOF and AA. EB, CO, CN, BOF, AA, OA and CA are natives of Nigeria. OA and CA are Nigeria's National TB Program representatives.

3. **How has funding been used to support the local research team?**

The study funded SHOPS Plus expenses related to the patient pathway surveys and interviews data collection and cleaning, staff time for research support, and expenses for disseminating results to local stakeholders.

4. **How are research staff who conducted data collection acknowledged?**

Several SHOPS Plus and Abts Associates staff involved in managing the data collection and cleaning processes (LR, BOF, BJ, AA and EB) are included as co-authors. All other staff and contractors involved in the field work are acknowledged.

5. **Do all members of the research partnership have access to study data?**

The data collected in this study was collected, cleaned and managed by local research staff. It was transmitted to team members at the Universities of McGill and Waterloo for analysis. All members of the research team have access to these data.

6. **How was data used to develop analytical skills within the partnership?**

Data analysis was performed by CO, LH, CN and MAHK. The team experimented with different ways of representing the pathways data and shared ideas with each other.

7. **How have research partners collaborated in interpreting study data?**

The research team worked together over several months to analyse and interpret the results from this research, with frequent inputs from more senior academics. This process was crucial in ensuring that the interpretation and contextualizing of the findings were true to the local context.

8. **How were research partners supported to develop writing skills?**

The research team responsible for analysing, interpreting and writing this report were predominantly early career researchers, composed of research assistants, graduate students and a then postdoctoral fellow. All authors made contributions to the manuscript.

9. **How will research products be shared to address local needs?**

The results from this study have been disseminated to local stakeholders including the National TB Program and representatives from TB program managers. Additionally, this manuscript will be published as open access.

10. **How is the leadership, contribution and ownership of this work by LMIC researchers recognized within the authorship?**

Coauthors BOF, OCA, AA and CA are LMIC researchers. Local staff within the SHOPS Plus project are acknowledged.

11. **How have early career researchers across the partnership been included within the authorship team?**

The vast majority of coauthors are early career researchers, including from LMICs (CO, LH, NA, MAHK, AA, CN, BOF, AA, and OCA).

12. **How has gender balance been addressed within the authorship?**

Eight authors are female, and six authors are male.

13. **How has the project contributed to improvements in local infrastructure?**

This project has not directly contributed to improvements in local infrastructure.

14. **What safeguarding procedures were used to protect local study participants and researchers?**

All field staff were trained and study instruments pilot-tested to ensure data quality. Collected data were anonymized by field staff before been shared with the rest of the research team. To protect patients and field staff from potential COVID-19 exposure, several precautionary measures were put in place. Prior to the start of survey implementation, a number of precautionary measures against COVID-19 were put in place. During training, temperature screening was carried out on the personnel before admittance into the training hall. The wearing of facemasks was enforced throughout the training period. Also, alcohol-based hand sanitizers were made available to the participants.

Appendix 4: Characteristics of respondents and non-respondents

		Patient that consented N=180	Patient that declined N=80	Overall N=260
**State**	Kano	90 (50.0%)	50 (62.5%)	140 (53.8%)
	Lagos	90 (50.0%)	30 (37.5%)	120 (46.2%)
**Gender**	Female	70 (38.9%)	30 (37.5%)	100 (38.5%)
	Male	110 (61.1%)	50 (62.5%)	160 (61.5%)
**Age Group**	18-24	24 (13.3%)	12 (15.0%)	36 (13.8%)
	25-34	63 (35.0%)	24 (30.0%)	87 (33.5%)
	35-44	50 (27.8%)	22 (27.5%)	72 (27.7%)
	45-54	21 (11.7%)	7 (8.8%)	28 (10.8%)
	55+	22 (12.2%)	15 (18.8%)	37 (14.2%)
**TB Status**	TB negative	90 (50.0%)	43 (53.8%)	133 (51.2%)
	TB positive	90 (50.0%)	33 (41.2%)	123 (47.3%)
	Missing	0 (0%)	4 (5.0%)	4 (1.5%)

Appendix 5 – Average number of encounters by type of provider seen first

Initial Provider contact	Total number of individuals	Mean Number of Encounters (SD)	Median (Min, Max)
Private hospital or nursing home	79	1.10 (0.304)	1.00 [1.00, 2.00]
Community Pharmacy or Medicine Vendor	57	2.35 (0.612)	2.00 [2.00, 5.00]
Public Hospital	26	2.31 (0.618)	2.00 [2.00, 4.00]
Public outpatient clinic	6	3.00 (1.265)	2.50 [2.00, 5.00]
Private laboratory	4	2.00 (0.00)	2.00 [2.00, 2.00]
Community Health Worker/CHEW	3	2.67 (0.577)	3.00 [2.00, 3.00]
Private outpatient clinic, health centre, or dispensary	3	3.33 (0.577)	3.00 [3.00, 4.00]
Traditional healers	1	3.00 (NA)	3.00 [3.00, 3.00]
Other, specify	1	4.00 (NA)	4.00 [4.00, 4.00]
TOTAL	180	1.84 (0.864)	2.00 [1.00, 5.00]

Appendix 6 - Selected qualitative themes and example quotes

Theme #	Theme Description	Codes	Sample Quote
1	Delays	Self-medication	"*Yes, it was up to a year, it was a gradual thing. I treated typhoid and malaria because that has always been the major sickness I usually have and when I treat, it subsides, and I'll think I'm okay until it crawls back again*," (#6, Female, TB +, Lagos).
		Self-medication/ Symptom minimization	"*I just thought it's the normal cough caused by* [*cold*] *and which makes me to be taking different cough syrups to be better, but it didn't work"* (#10, Female, TB -, Kano).
		Symptom minimization	"*…for instance, you can't just get up and visit the doctor in a haste because of headache. It is just like one going to the farm and feeling the hot sun but knowing that in a matter of time you will feel relief from the effect of the sun. And that was why I spent some few days before visiting the chemist*." (#5, Male, TB +, Kano).
		Symptom minimization /Misdiagnosis	"*I was coughing blood. I didn't take it seriously … when I came home, my dad asked me to take herbs … it persisted, so my mum took me to the chemist who gave me drugs to take for 10 days. I felt if I completed the 10 days it would stop. After completing it, it didn't stop,*" (Male, TB Positive, Lagos). (#9, Male, TB +, Lagos).
		Misdiagnosis	"*…Then we didn't know what the sickness was, we didn't know what type of cough it was. I went to chemist, I was tested and given some drugs for catarrh and I felt relieve after taking it but whenever the medicine is finished the cough will be back"* (Female, TB Positive, Kano). (#7, Female, TB +, Kano).
		Same day treatment	*"I was diagnosed at that time … in the hospital when we came back …it was all done here, after the test and checking, drugs was given to us on the same day. …"* (Female, TB Positive, Kano). (#4, Female, TB +, Kano).
		Same day treatment	"*I was told to come back the same day for the test result ... When I came back, I was told about the TB and that I needed to commence treatment. I was asked some questions... how many people I stayed with, if where I stayed had ventilation .... I was given the drugs to take ... When I started treatment, my chest pain started reducing*" (#9, Male, TB +, Lagos). (#9, Male, TB +, Lagos).
2	Pathways	Symptoms progressing/ misdiagnosis	"*when it started, I thought, it was just a mild cough, that was why I used cough syrup. It was later, when I started coughing out blood, that I now thought that this has passed normal cough because it was so serious, that was why I went to the hospital, they now referred me [from one*

			*public hospital to another] and from there, we know came here [private hospital]" (*Male, TB positive, Lagos).<br>(#5, Male, TB +, Lagos).
		Symptoms progressing	"*…it was excessive coughing that had been disturbing me; and I use to go and collect drugs … but that didn't change anything, so I took drugs for a long time*" (Male, TB positive, Kano).<br>(#2, Male, TB +, Kano).
3	Choice of initial provider	Proximity and convenience	"*The chemist is closest to us, without spending on transportation or ... other services. Chemist services are daily and constant compared to the hospital where you will be given time to see the doctors and they rarely operate at nigh*t." (#10, Female, TB -, Kano).<br>(#10, Female, TB -, Kano).
		Recommendations of family/friend	*"The whole process took long because there was no sign of cough just that it was eating me ... So, … when I travelled home, a matron from my family … asked me if I'd gone for test. I told her I had gone for series of tests and she told me to go for TB test which made me laugh and I told her …I clearly wasn't coughing. … she told me it doesn't matter because TB has a way of manifesting by eating one up internally …. When I got back to Lagos, someone else told me the same thing which made me go for test at the Lab… Not until when I came here and the doctor prescribed 3 tests which I did and that was when I started coughing which was increasing gradually … up to a week and at a point I started coughing out blood".*<br>(#6, Female, TB +, Lagos).
4	Use of multiple providers	Misdiagnosis, recommendation of friend	"*I went to the chemist [after my symptoms started] ... to a private doctor for cough syrup and tablets. Then ...to a public hospital during pandemic, there was long queue...I took herbs... went to another hospital. This boy that works in a hospital asked if I had conducted a test … he then said I should go to the lab with a note he has written.* " (#5, Male, TB +, Kano).<br>(#5, Male, TB +, Kano).
		Misdiagnosis, recommendation of friend	"*It has been the long I had the symptoms. My mum, we were thinking that it was malaria or fever, so I've been using malaria drugs since February, because our family nurse told us that it was malaria, so she was just giving me injections, and drugs and I did not like it. We went to the hospital by March and they told me that it was fever, so I was also given injections, and drugs … I told them that I was coughing and having chest pain, but the drugs they gave me… were just for malaria. I was complaining to the doctor ...that I was coughing. I did blood tests too but the drugs they were giving me, it was actually not relieving, so I told my doctor in our church... It was him that referred me to … to go for a x-ray...because my mum once had TB,*

			maybe it could be TB, he guessed... I went for x-ray…and it was TB and the sputum test came out too, it was TB". (#1, Female, TB +, Lagos).
5	Impact of COVID	Fear of diagnosis	*I will be thinking that if I have this malaria, it's better I go to the chemist to buy the anti-malaria drug than going to the hospital where I will be told that I have COVID-19 just because I have malaria symptoms*. (Female TB positive, Lagos)<br>(#1, Female, TB +, Lagos).
		Provider attitude, overburdened facilities, and infection control mandates	"*Actually, there are a lot of differences you will notice. During the COVID-19 period, the doctors around will be harassing people without considering their condition. … And those COVID-19 protocols are some of the serious challenges we faced. At that time, they will tell you to use face mask, use hand sanitizer and social distancing… And there was congestion, too much death rate. Actually COVID-19 has affected my access to health care services a lot*".<br>(#10, Female, TB -, Kano).
		Movement restrictions, infection control mandates, provider attitude	"*As villagers, we don't know much about COVID-19. It … affected me during lockdown that I couldn't get access to the hospital. My major challenge was during the lockdown. I don't like that covid-19 protocols, wearing of face masks does not allow me to breath well, that social distancing. And above all even the healthcare providers are afraid to attend to us because COVID-19 and TB are communicable diseases and have similar symptoms".*<br>(#9, Male, TB +, Kano).
